# Provision of foot health services for people with rheumatoid arthritis in New South Wales: a web-based survey of local podiatrists

**DOI:** 10.1186/1757-1146-6-35

**Published:** 2013-08-26

**Authors:** Gordon J Hendry, Kathryn A Gibson, Kevin Pile, Luke Taylor, Verona du Toit, Joshua Burns, Keith Rome

**Affiliations:** 1 School of Science & Health, University of Western Sydney, Penrith, NSW, Australia; 2 Institute for Applied Health Research, Glasgow Caledonian University, Glasgow G4 0BA, UK; 3 Department of Rheumatology, Liverpool Hospital, Sydney, NSW, Australia; 4 Campbelltown Hospital, School of Medicine University of Western Sydney, Campbelltown, NSW, Australia; 5 Podiatry Department, Camden & Campbelltown Hospital, South Western Sydney Local Health District, Sydney, NSW, Australia; 6 School of Medicine, University of Western Sydney, Penrith, NSW, Australia; 7 The University of Sydney and Sydney Children’s Hospitals Network (Randwick and Westmead), Sydney, NSW, Australia; 8 Department of Podiatry, Health and Rehabilitation Research Institute, School of Rehabilitation & Occupational Studies, AUT, Auckland, New Zealand

**Keywords:** Rheumatoid arthritis, Foot health, Podiatry, Footwear, Care access, Web survey

## Abstract

**Background:**

It is unclear if podiatric foot care for people with rheumatoid arthritis (RA) in New South Wales (NSW) meets current clinical recommendations. The objective of this study was to survey podiatrists’ perceptions of the nature of podiatric foot care provision for people who have RA in NSW.

**Methods:**

An anonymous, cross-sectional survey with a web-based questionnaire was conducted. The survey questionnaire was developed according to clinical experience and current foot care recommendations. State registered podiatrists practising in the state of NSW were invited to participate. The survey link was distributed initially via email to members of the Australian Podiatry Association (NSW), and distributed further through snowballing techniques using professional networks. Data was analysed to assess significant associations between adherence to clinical practice guidelines, and private/public podiatry practices.

**Results:**

86 podiatrists participated in the survey (78% from private practice, 22% from public practice). Respondents largely did not adhere to formal guidelines to manage their patients (88%). Only one respondent offered a dedicated service for patients with RA. Respondents indicated that the primary mode of accessing podiatry was by self-referral (68%). Significant variation was observed regarding access to disease and foot specific assessments and treatment strategies. Assessment methods such as administration of patient reported outcome measures, vascular and neurological assessments were not conducted by all respondents. Similarly, routine foot care strategies such as prescription of foot orthoses, foot health advice and footwear were not employed by all respondents.

**Conclusions:**

The results identified issues in foot care provision which should be explored through further research. Foot care provision in NSW does not appear to meet the current recommended standards for the management of foot problems in people who have RA. Improvements to foot care could be undertaken in terms of providing better access to examination techniques and treatment strategies that are recommended by evidence based treatment paradigms.

## Background

Rheumatoid arthritis affects approximately 2.5% of the Australian population [1] and commonly results in foot problems including joint pain, stiffness, and deformities [2]. There is emerging evidence of unmet need for, and several barriers to appropriate foot care provision in New South Wales (NSW) [3]. It is unclear what specific foot care components are currently available to patients with RA who gain access to podiatry services. Furthermore, it is unclear whether podiatric foot care that is provided meets current evidence based recommendations [1,4-8] with regards to the assessment and management of disease-related foot problems in people with RA. The most recently published guidelines provide both specialist and non-specialist podiatrists with essential and ‘gold standard’ recommendations for the management of people with RA-related foot problems [8].

The podiatric management of people with RA has evolved recently with a greater emphasis being placed upon rapid access to expert-led and integrated multidisciplinary rheumatology teams for rigorous examinations and personalised targeted therapies based on treat-to-target principles [9,10]. Briefly, the treat-to-target approach is where the goal of therapeutic intervention is to achieve an acceptable level of an outcome of importance (such as low levels of inflammatory disease activity) whereby further damage or deterioration will not occur [9].

There is evidence that integrated multidisciplinary foot care for people who have RA is being implemented elsewhere such as the UK, The Netherlands and New Zealand and preliminary evidence of improvements in patient outcomes has been demonstrated through prospective cohort studies [10-12]. However there is evidence from the UK suggesting that regional variation in foot health services is high [13], and that there may be an insufficient number of specialist podiatrists to meet the complex needs of the RA population [8]. As such, there is a significant need to describe the current nature of foot care provision for people with RA in NSW, in order to determine whether or not it meets modern day recommendations.

Accordingly, the primary aim of this study was to survey podiatrists’ perceptions of the current nature of podiatric foot care provision for people who have RA in NSW. The secondary aims of this study were to determine if the level of adherence to clinical practice guidelines for the management of foot problems in RA are associated with access to specific components of foot health services, and evaluate whether public or private podiatry practices are associated with access to specific components of foot health services.

## Methods

### Design

A web-based questionnaire was conducted. The survey was available for completion for 11 months, from the 10^th^ January to the 16^th^ December 2012.

### Participants

Participants were recruited using convenience and snowball sampling techniques. Podiatrists practising in the state of NSW were invited to participate via an invitation email from the Australian Podiatry Association (NSW). To maximise the response rate, podiatrist members of the Australasian Podiatric Rheumatology Special Interest Group (APRSIG) who were based in NSW were invited to take part via an advertisement on the APRSIG website [14]. The survey was also promoted at a Sydney Local Health District Podiatry Education/Continual Professional Development Day, and a number of continuing professional development events held at the Australian Podiatry Association (NSW) rooms. Qualified podiatrists based in NSW who were external clinical supervisors for the Podiatric Medicine program (undergraduate and direct entry masters), University of Western Sydney, School of Science and Health, were also invited to take part. According to the Podiatry Board of Australia Podiatry Registrant Data [15], approximately 992 podiatrists were registered to practice in 2013.

Ethical approval was obtained from the South Western Sydney Local Health District and the University of Western Sydney Research Ethics Committees. As outlined in the participant information sheet instructions, consent was assumed if participants followed the web-link and completed the survey. Written informed consent was not sought from participants in order to ensure anonymity.

### Data gathering

The survey questionnaire was adapted from the original work by Redmond et al [13] to determine the availability of specific foot health service components for RA patients in NSW. Briefly, the original questionnaire content which was aimed at rheumatologists was re-worded in order to survey podiatrists in NSW. The original questionnaire content regarding assessment methods and treatment strategies was reviewed and additions were made in line with current foot care recommendations (i.e. inclusion of musculoskeletal ultrasound as an assessment method for inflammatory foot disease). The survey was subject to pilot testing by all co-authors to ensure the relevance of the questions, and the final questionnaire was amended according to feedback. Three iterative revisions were conducted by the research team and these were based upon previous research [13], clinical experience, and current foot care recommendations [8,10] (see Additional file 1). The survey was created using web-based questionnaire software (Survey Monkey®) [16] and distributed via electronic web link. Using the Survey Monkey® software survey functions, only fully completed questionnaires were included in the analysis. Moreover only one response was permitted per computer (IP address tracking) to restrict submission of multiple invalid duplicate responses from individual participants.

### Data analysis

Data were analysed using SPSS v.19.0 (SPSS, Chicago, IL, USA). The primary analysis was descriptive statistics. Secondary analysis was conducted using cross-tabulation and chi-square and Fisher’s exact test statistics. Categorical data regarding access to various aspects of foot care were cross-tabulated according to two categorical dependent variables derived from two questions in the survey; “Do you use any formal guidelines/protocols for the management of patients with RA who have foot problems?”, and “Is your clinic a public or a private clinic?”. Pearson’s chi-square and Fisher’s exact test statistics with corresponding odds ratios (OR) were performed to determine the strength of any significant associations between: RA foot care guidelines use and access to foot care attributes, and between publicly or privately employed podiatrists and access to foot care attributes. Where cell frequencies in 2 x 2 cross-tabulated contingency tables were less than 5, Fisher’s exact test was preferred. In all tests, *p* < 0.05 was considered to indicate statistical significance.

## Results

### Respondents podiatry practice characteristics

**Figure 1:**
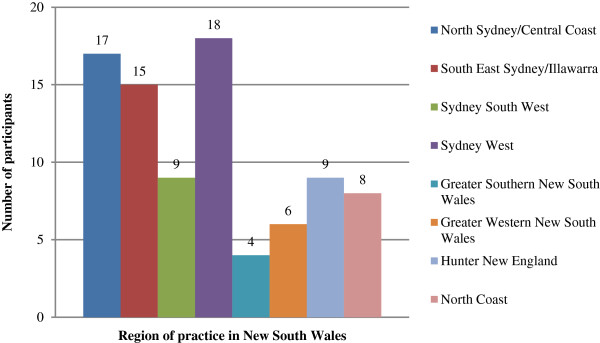
Bar chart indicating the distribution of respondents’ regions of practice in NSW.

**Figure 2:**
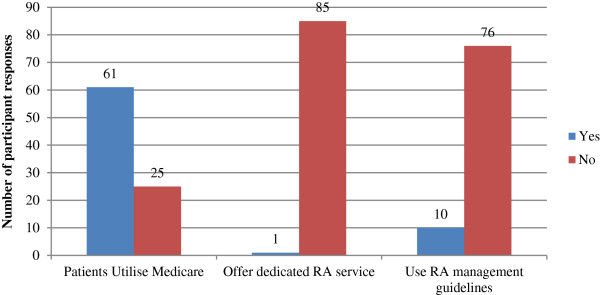
Bar chart indicating what foot care provision was available for people with RA.

**Figure 3:**
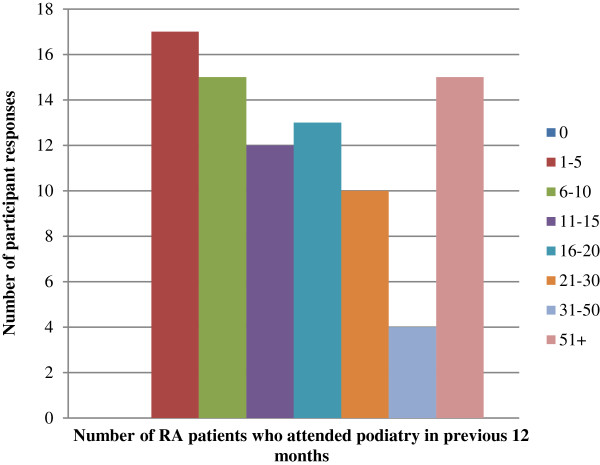
Bar chart describing respondents’ responses to the question “How many patients with RA attended your practice for foot care in the previous 12 months?”.

**Figure 4:**
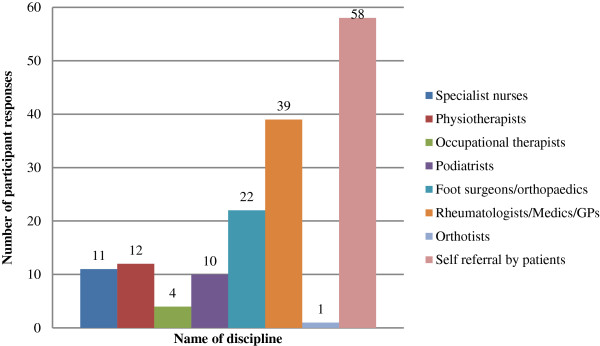
Bar chart indicating participants’ responses concerning the health professionals that refer patients with rheumatoid arthritis to podiatry.

Descriptive data regarding podiatry practice characteristics are presented in Figures 1, 2, 3 and 4. 86 podiatrists who practised in 8 regions of NSW participated in the survey, with 51 (69%) indicating they practised within Greater Sydney region boundaries (North Sydney, South East Sydney, Sydney South West, and Sydney West). Sixty-seven respondents (78%) worked predominantly in private practices, while 19 (22%) worked predominantly in publicly funded podiatry practices. Only 1 of 86 respondents indicated that they offered a dedicated service specifically for patients with RA (Figure 2). Sixty-one respondents (71%) indicated that patients with RA utilised the Medicare Enhanced Primary Care programme (a programme that permits patients with certain chronic health conditions to receive partial reimbursement of costs for up to five appointments with allied health professionals) to attend their podiatry clinic for foot care; and only 10 respondents (12%) indicated that they use formal clinical practice guidelines for managing their RA patients (Figure 2). All respondents indicated that at least 1-5 patients with RA attended their practice within the previous 12 months, however responses were variable (Figure 3). Self-referral by patients was the most frequently reported method of access to podiatry (68%). Referral by medical disciplines (rheumatologists/doctors/GPs) was also frequently reported (46%) while referrals from other health professional disciplines appeared to be less common (Figure 4).

### Access to recommended foot examination/assessment characteristics

**Figure 5:**
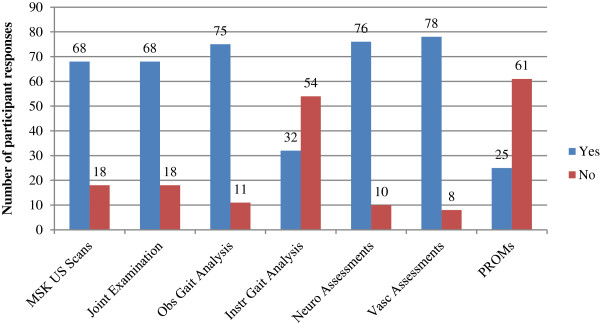
Bar chart indicating the number of ‘yes/no’ responses to the question regarding foot health assessments “Do your patients have adequate access to services providing for these needs:-”.

Descriptive data regarding access to specific foot examination/assessment characteristics are presented in Figure 5. Generally respondents indicated that RA patients could access most examinations/assessment methods at their practices. However, 61 (71%) respondents indicated that they did not administer patient-reported outcome measures (PROMs) to monitor their RA patients. Fifty-four respondents (63%) also indicated that there was no access to instrumented gait analysis. Adequate access to examinations of individual foot joints for tenderness and swelling, and musculoskeletal ultrasound scans were not available for 18 (21%) respondents’ practices respectively.

### Access to recommended foot care/treatment characteristics

**Figure 6:**
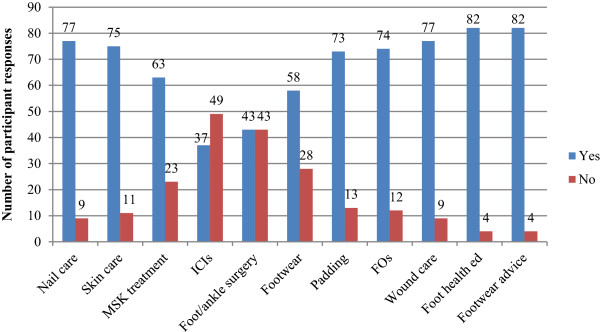
Bar chart indicating the number of ‘yes/no’ responses to the question regarding foot health treatments “Do your patients have adequate access to services providing for these needs:-”.

Descriptive data regarding access to specific foot care/treatment characteristics are presented in Figure 6. The majority of participants indicated that their RA patients would have access to routine foot care components such as nail care. However several respondents indicated patients with RA would not have access to more specialised foot care/treatments at their practice such as intra-articular corticosteroid injections and customised footwear.

### Guidelines use and access to foot assessments/examinations

We found significant association between guideline use and administration of PROMs to monitor outcomes of RA patients (*p* = 0.001, OR: 13.9). There was also a significant association between guideline use and access to instrumented gait analysis (*p* = 0.028, OR: 4.8). No other significant associations (*p* > 0.05) were observed (Table 1).

**Table 1:** Table outlining associations between adherence to guidelines and public/podiatry, with access to components of foot care (assessments/examinations)

**Access to foot care variable**	**Guidelines yes**	**Guidelines no**	**OR**	***p***^**†**^	**Public**	**Private**	**OR**	***p***^**†**^
US scan yes, n (%)	7 (70)	61 (80.3)	0.57	0.348	12 (63.2)	56 (83.6)	0.34	0.058
US scan no, n (%)	3 (30)	15 (19.7)	-	-	7 (36.8)	11(16.4)	-	-
Joints exam yes, n (%)	8 (80)	60 (78.9)	1.07	0.652	14 (73.7)	54 (80.6)	0.67	0.358
Joints exam no, n (%)	2 (20)	16 (21.1)	-	-	5 (26.3)	13 (19.4)	-	-
Ob gait analysis yes, n (%)	8 (80)	67 (88.2)	0.54	0.376	12 (63.2)	63 (94.0)	0.11	**0.002**
Ob gait analysis no, n (%)	2 (20)	9 (11.8)	-	-	7 (36.8)	4 (6.0)	-	-
Ins gait analysis yes, n (%)	7 (70)	25 (32.9)	4.76	**0.028**	4 (21.1)	28 (41.8)	0.37	0.081
Ins gait analysis no, n (%)	3 (30)	51 (67.1)	-	-	15 (78.9)	39 (58.2)	-	-
Neuro yes, n (%)	9 (90)	67 (88.2)	1.21	0.672	18 (94.7)	58 (86.6)	2.79	0.299
Neuro no, n (%)	1 (10)	9 (11.8)	-	-	1 (5.3)	9 (5.3)	-	-
Vascular yes, n (%)	9 (90)	69 (90.8)	0.91	0.645	18 (94.7)	60 (89.6)	2.1	0.434
Vascular no, n (%)	1 (10)	7 (9.2)	-	-	1 (5.3)	7 (10.4)	-	-
PROMs yes, n (%)	8 (80)	17 (22.4)	13.9	**0.001**	3 (15.8)	22 (32.8)	0.38	0.121
PROMs no, n (%)	2 (20)	59 (77.6)	-	-	16 (84.2)	45 (67.2)	-	-

^†^*P* values are based on two-sided chi-square test or Fisher’s exact test; **bold** text indicates *p* < 0.05.

*Abbreviations: OR* odds ratio, *US* ultrasound, *Ob* observational, *Ins* intrsumented, *Neuro* neurological assessment, *Vascular* vascular assessment, *PROMs* patient-reported outcome measures.

### Public/private podiatry practices and access to foot assessments/examinations

Table 1 demonstrates a significant association was observed between access to observational gait analysis and private practice (*p* = 0.002, OR 0.11). No other significant associations were observed (*p* > 0.05).

### Guidelines use and access to foot care

Detailed associations between guideline use and foot care access are presented in Table 2. No statistically significant associations were observed (*p* > 0.05).

**Table 2:** Table outlining associations between adherence to guidelines and public/podiatry, with access to components of foot care (treatments)

**Foot care variable**	**Guidelines yes**	**Guidelines no**	**OR**	***p***^**†**^	**Public**	**Private**	**OR**	***p***^**†**^
Nail care yes, n (%)	9 (90)	68 (89.5)	1.06	0.720	15 (78.9)	62 (92.5)	0.30	0.104
Nail care no, n (%)	1 (10)	8 (10.5)	-	-	4 (21.1)	5 (7.5)		-
Skin care yes, n (%)	9 (90)	66 (86.8)	1.36	0.624	15 (78.9)	60 (89.6)	0.44	0.198
Skin care no, n (%)	1 (10)	10 (13.2)	-	-	4 (21.1)	7 (10.4)		-
MSK treatment yes, n (%)	8 (80)	55 (72.4)	1.53	0.467	14 (73.7)	49 (73.1)	1.09	0.606
MSK treatment no, n (%)	2 (20)	21 (27.6)	-	-	5 (26.3)	19 (26.9)		-
ICIs yes, n (%)	5 (50)	32 (42.1)	1.38	0.442	7 (36.8)	30 (44.8)	0.72	0.364
ICIs no, n (%)	5 (50)	44 (57.9)	-	-	12 (63.2)	37 (55.2)		-
Foot surgery yes, n (%)	6 (60)	37 (43.0)	1.58	0.369	10 (52.6)	33 (49.3)	0.60	0.500
Foot surgery no, n (%)	4 (40)	39 (48.7)	-	-	9 (47.4)	34 (50.7)		-
Footwear yes, n (%)	8 (80)	50 (65.8)	2.08	0.304	15 (78.9)	43 (64.2)	2.09	0.176
Footwear no, n (%)	2 (20)	26 (34.2)	-	-	4 (21.1)	24 (35.8)		-
Padding yes, n (%)	8 (80)	65 (85.5)	0.68	0.468	15 (78.9)	58 (86.6)	0.58	0.311
Padding no, n (%)	2 (20)	11 (14.5)	-	-	4 (21.1)	9 (13.4)		-
FOs yes, n (%)	8 (80)	66 (86.8)	1.65	0.423	13 (68.4)	61 (91.0)	0.21	**0.021**
FOs no, n (%)	2 (20)	10 (13.2)	-	-	6 (31.6)	6 (9.0)		-
Wound care yes, n (%)	9 (90)	68 (89.5)	1.06	0.720	19 (100)	58 (86.6)	2.94	0.093
Wound care no, n (%)	1 (10)	8 (10.5)	-	-	0 (0)	9 (13.4)		-
Foot health ed yes, n (%)	10 (100)	72 (94.7)	0.56	0.604	18 (94.7)	64 (95.5)	0.84	0.639
Foot health ed no, n (%)	0 (0)	4 (5.3)	-	-	1 (5.3)	3 (4.5)		-
Footwear adv yes, n (%)	10 (100)	72 (94.7)	0.56	0.604	17 (89.5)	65 (97.0)	0.26	0.210
Footwear adv no, n (%)	0 (0)	4 (5.3)	-	-	2 (10.5)	2 (3.0)		-

^†^*P* values are based on two-sided chi-square test or Fisher’s exact test; **bold** text indicates *p* < 0.05. *Abbreviations: OR* odds ratio, *MSK* musculoskeletal, *ICIs* intra-articular steroid injection, *FOs* foot orthoses, *Ed* education, *Adv* advice.

### Private/public podiatry practices and access to foot care

Detailed associations between public/private podiatry practice and foot care access are presented in Table 2. A significant association was observed between access to foot orthoses and private practice (*p* = 0.021, OR 0.21). No other significant associations were observed (*p* > 0.05).

## Discussion

This preliminary study has described the current nature of podiatric foot care provision for people who have RA in NSW by eliciting data on the availability of foot health service components from a sample of local podiatrists. Several important aspects of service provision were identified including the lack of dedicated/integrated podiatric rheumatology services for treatment of disease-related foot problems in people with RA. This is an important finding as our recent research has demonstrated that there may be several barriers to, and an unmet need for appropriate foot care in NSW [3]. Dedicated specialist foot care services have been strongly recommended for people with RA [1,4-8,10,13,17,18]. There is emerging evidence that integrated specialist foot care services can improve clinical outcomes in people with RA [11,12]. Integration of podiatry services within rheumatology centres in NSW could resolve the unmet needs of people with RA in this region of Australia.

There appears to be a lack of awareness of clinical practice guidelines for the management of foot problems in RA, or indeed a poor level of adherence to these guidelines. Similar findings have been demonstrated in the UK, with non-specialist podiatrists being less likely to use RA foot care guidelines to inform their podiatric practice [19]. The vast majority of respondents (88%) in this study indicated that they do not use formal guidelines to inform their management of patients with RA who have foot problems. It is unclear why the majority of respondents reported that they do not use clinical practice guidelines; however it is acknowledged that current guidelines for podiatric management of RA were developed in the UK for podiatrists to follow within the context of the UK National Health Service [4-8]. As such it is possible that podiatrists in Australia would not consider UK foot care guidelines relevant to the Australian health care context, thus resulting in a poor uptake of these guidelines. Other potential barriers to the use of guidelines reported in the literature include perceptions of a threat to practitioner autonomy, complaints regarding overly lengthy and complex information included within guidelines, and perceptions of irrelevance to practitioners’ clinical practice [19]. Further work is required to increase awareness of clinical practice guidelines for management of foot problems in RA.

Currently, little is known about referral pathways to podiatry for RA patients. An important finding in the current study was that the main route to podiatric foot care was by patient self-referral. A recent study has reported that patients with RA are predominantly responsible for choosing to access foot care service [20]. However, there is growing evidence to suggest that patients with RA may not be suitably equipped with the knowledge and understanding of their disease-related foot problems and therefore do not undertake timely self-referral to podiatry [3,20-22]. Therefore, there appears to be a significant need for increased awareness and uptake of rapid foot care referral guidelines amongst multidisciplinary rheumatology teams and podiatry services [10]. The main referring physicians of RA patients to podiatry identified in this survey were rheumatologists/doctors/general practitioners (GPs). This is perhaps unsurprising as Australian general practice guidelines for the management of RA stipulate that access to appropriate foot care should be strongly supported by GPs [1].

The concept of tight disease control and monitoring has been identified as an important component of patient-centred, personalised and outcome driven care for patients with RA [10]. Several well–validated patient-reported outcomes measures (PROMs) such as the Foot Impact Scale (FIS) for RA have been developed and are widely available for measuring foot-related impairments and disability [23,24]. However, the majority of respondents indicated that they do not use PROMs to monitor outcomes of RA patients in their practices. This may be of concern because there is a need for objective evaluation of disease-specific foot-related outcomes in order to optimise the effect of interventions [23]. It is possible that there are perceived barriers to the use of PROMs in everyday clinical practice such as time burden to administer and interpret. However it is usually recommended that patients are invited to complete PROMs in the waiting area prior to their podiatry appointment. A significant association (*p* = 0.001, OR 13.9) was observed between conformance to clinical practice guidelines and use of PROMs to monitor outcomes in people with RA. Indeed the use of PROMs are strongly advised in RA foot care guidelines [1,4-8].

Musculoskeletal ultrasound is increasingly being used by UK-based podiatrists in extended scope roles as it is a superior method to clinical examination for detecting and monitoring disease activity in the inflammatory joint diseases [25-27]. At present it is unclear what training is available to support the podiatry workforce in NSW and Australia generally. Nevertheless the majority of respondents (79%) indicated that their RA patients would have access to musculoskeletal ultrasound scans. An association approaching statistical significance (*p* = 0.058, OR 0.34) was observed between private podiatry practices and access to musculoskeletal ultrasound, suggesting that access to musculoskeletal ultrasound may not be as readily available through public podiatry services. The reasons for this are unknown, however it is possible that there are better referral pathways between podiatrists and sonographers in the private sector.

Several instrumented gait analysis techniques have been successfully employed to objectively quantify foot function in people who have RA [23,28,29]. As a result, instrumented gait analyses such as plantar pressure and spatio-temporal measurements have been included in recommendations for extended outcome datasets for monitoring foot and ankle disease in RA [10]. It is acknowledged that there are a variety of instrumented gait analysis methods that will have varying levels of utility for measuring gait parameters in the RA population. For the purposes of this survey, we did not specifically define instrumented gait analysis according to specific methods. As such patients may have had access to various different gait analysis methods. Historically instrumented gait analysis techniques have been used predominantly for research purposes through academic-clinical partnerships. However, there is emerging evidence from this survey that instrumented gait analysis may be more widely available to people with RA. Over one-third of respondents indicated that RA patients would have access to instrumented gait analysis through their practice. There was a significant association between conformance with clinical practice guidelines and access to instrumented gait analysis (*p* = 0.028, OR 4.8). The reason for this finding is unclear; however it is possible that those who adhere to guidelines may be more aware of the potential benefits of objective and comprehensive assessments of gait in people who have RA.

Approximately 10% of respondents indicated that people with RA would not have access to neurological or vascular assessments through their practice. Previous research has demonstrated that people with RA may be at risk of developing peripheral vascular disease and a loss of protective sensation [30,31]. Indeed, recently published guidelines have recommended that vascular examinations (including assessments of intermittent claudication/rest pain, vasculitis, pulses using Doppler ultrasound), and neurological examinations (including assessments of sensory loss and nerve entrapment/compression), should be conducted as part of the core assessments of patients with RA [8].

In terms of treatment components of podiatric care for people with RA, core treatments such as nail care, foot orthoses, and footwear advice were generally offered, but not by all respondents. Access to provision of footwear was not as readily available to RA patients, with one-third of respondents indicating their patients would not have access through their practice. There is evidence to suggest that specialist footwear can improve clinical outcomes in RA [32]. However, there is a paucity of research outlining the availability of services offering customised/bespoke footwear for people with RA in Australia. Furthermore, there appears to be a lack of guidance with regards to which patients should receive customised footwear and which specific protective footwear features should be incorporated therein. Researchers in Australia have recently recommended that health professionals should be aware of state- and territory-based equipment funding schemes that can provide financial assistance to eligible patients who require footwear [33]. In NSW, a relatively new scheme known as Enable NSW now offers equipment (including footwear and orthoses) to eligible state residents who have a permanent or long-term disability [34]. It is possible that this scheme may result in greater access to appropriate footwear to those RA patients who have financial difficulties.

A small proportion of participants (14%) indicated that their RA patients would not have access to foot orthoses (FOs) through their practice. This is a surprising finding as a recent systematic review with meta-analysis has demonstrated that FOs can significantly reduce foot pain in early RA [35]. It is possible that some podiatrists may not have access to certain published articles due to subscription-only access restrictions, particularly those who work predominantly in private practice. Moreover podiatrists have previously identified that they lacked time in clinical practice to read any guidelines [19]. However, a significant association was found between private practice and access to FOs (*p* = 0.021, OR 0.21), suggesting greater odds of patients accessing FOs through private practice compared to public podiatry services. At present it is unclear why FOs would be less accessible in public podiatry clinics and further research is required to investigate this finding.

The majority of respondents (57%) indicated that intra-articular corticosteroid injections (ICIs) for the foot and ankle were not accessible through their practices. ICIs are effective in arresting localised inflammation and relieving painful symptoms in the short-to-medium term in people with RA [36,37] and may be a valuable treatment option for suitably trained podiatrists. It is acknowledged that significant training has to be undertaken in accordance with the Podiatry Board of Australia guidelines for Endorsement for Scheduled Medicines before podiatrists qualify for prescribing rights [38]. However several corticosteroid preparations including triamcinolone (injectable solution) are available to podiatrists who complete the required training [38]. As such, an increase in the number of podiatrists who are qualified to administer ICIs amongst the NSW workforce could improve foot-related outcomes in people who have RA and local disease activity.

Due to the web-based design of this survey and the technique adopted to distribute the survey link, we were unable to calculate a response rate. However the survey response count of 86 participants represents approximately 8.7% of the podiatry workforce in NSW [15]. While this represents a small proportion of practising podiatrists in NSW, the proportion of respondents working in private and public podiatry practices is similar to that outlined in the NSW Podiatry Workforce Report [39]. This suggests that our sample may have been representative of the population of NSW podiatrists. Web surveys have several advantages such as shorter transmitting time, lower costs, and less data entry time [40]. However, a recent meta-analysis has estimated that response rates in web-based surveys are on average approximately 11% less than that of other survey methods [41]. Moreover, the data from this study do not necessarily represent ‘actual’ care access, but local podiatrists’ opinions/perceptions of access to foot care components at their predominant practice. As such this data may be subject to response and recall bias. It is likely that accurate data regarding foot care access and service provision in NSW could be elicited using prospective cohort designs such as those conducted in The Netherlands and the UK recently [42,43].

## Conclusions

This study has provided a preliminary description of foot care provision for people who have RA in NSW, and has identified several potential shortfalls in foot care provision which should be explored through further research. It appears as though several podiatry practices do not meet the current recommended standards of care for the management of foot problems in people who have RA. Improvements to foot care could be made in terms of providing better access to important assessments including patient-reported outcome measures, vascular and neurological examinations; and better access to appropriate treatments including foot orthoses, customised footwear, and intra-articular corticosteroid injections. An increase in availability and uptake of musculoskeletal ultrasound training as well as qualifications in scheduled medicines prescribing rights for podiatrists could lead to improvements in the foot care of RA patients through implementation of tight control of disease activity, prevention of foot-related disability, and personalised treatment plans. Integration of podiatry within rheumatology centres, and/or rapid access to expert-led multidisciplinary care teams including podiatry may lead to improvements in the outcomes of patients with RA and disease-related foot problems.

## Competing interests

The authors declare that they have no competing interests.

## Authors’ contributions

GJH conceived and executed the study protocol (with contributions from KAG, KP, VdT, JB and KR). All co-authors contributed to the design of the survey questionnaire. GJH interpreted the findings with assistance from all co-authors. GJH drafted the manuscript and the final version was read and approved by all co-authors.

## Supplementary Material

Additional file 1 Podiatrist E-Survey Questionnaire. Click here for file
