# Supplementary material for: Provision of foot health services for people with rheumatoid arthritis in New South Wales: a web-based survey of local podiatrists
Source: J Foot Ankle Res. 2013 Aug 26;6:35. doi: 10.1186/1757-1146-6-35 (PMC3765430; doi:10.1186/1757-1146-6-35)
Supplement: Additional file 1 — Podiatrist E-Survey Questionnaire. [file 1757-1146-6-35-S1.docx]

Podiatrist E-Survey Questionnaire

1. In which region of New South Wales are you practicing? (drop down box with all regions)

Area Health Services in Metropolitan NSW

- 1. Northern Sydney/Central Coast
  2. South East Sydney/Illawarra
  3. Sydney South West
  4. Sydney West

Area Health Services in Rural NSW

- 1. Greater Southern
  2. Greater Western
  3. Hunter New England
  4. North Coast

1. How many patients who have rheumatoid arthritis have attended your clinic over the past 12 months? _________
2. Is your clinic a public or private clinic? __________
3. Which of the following disciplines currently refer patients with rheumatoid arthritis directly into your clinic? (please tick all that apply.)

| **Specialist nurses** | **Physio - therapists** | **Occupational therapists** | **Podiatrists** | **Foot surgeons , orthopaedics** | **Rheumatologists, medics** | **Orthotists** | **Self referral by patients** |
| --- | --- | --- | --- | --- | --- | --- | --- |
| [ ] | [ ] | [ ] | [ ] | [ ] | [ ] | [ ] |  |

1. Do you offer dedicated clinics specifically for providing foot health services for your patients who have rheumatoid arthritis? Y/N
2. Do you use any formal guidelines/protocols for the management of RA patients with foot problems? Y/N
3. Do your patients have adequate access to services providing for these needs?
4. Assistance with nail care Y/N
5. Reduction (debridement) of callus and corns Y/N
6. Management of musculoskeletal symptoms in the feet and ankles Y/N
7. Image guided intra-articular steroid injections in the feet and ankles for localised inflammation Y/N
8. Foot/ankle surgery
9. Do your patients have adequate access to these services?
10. Provision of footwear Y/N
11. Provision of insoles and padding Y/N
12. Provision of functional foot orthoses Y/N
13. Provision of wound care/debridement and dressings Y/N
14. Provision of foot health/ disease advice Y/N
15. Provision of footwear advice Y/N
16. Do your RA patients have adequate access to these assessments/examinations?
17. Musculoskeletal ultrasound scans Y/N
18. Examination of tender/swollen foot joints and soft tissues Y/N
19. Observational gait analysis Y/N
20. Instrumented gait analysis Y/N
21. Neurological assessments Y/N
22. Vascular assessments Y/N
23. Do you use any of the following to monitor outcomes of RA patients in your practice?
24. Leeds Foot Impact Scale
25. Manchester Foot Pain and Disability Questionnaire
26. Foot Health Status Questionnaire
27. American Academy of Orthopaedic Surgeons Lower Limb Outcomes Assessment Instruments: Foot and Ankle Module
28. Salford Rheumatoid Arthritis Foot Evaluation Instrument
29. Other, please specify_________________
